# Supplementary material for: Multi‐dataset identification of innovative feature genes and molecular mechanisms in keratoconus
Source: J Cell Mol Med. 2024 Sep 19;28(18):e70079. doi: 10.1111/jcmm.70079 (PMC11412914; doi:10.1111/jcmm.70079)
Supplement: Supplementary file 2 — Material S2. [file JCMM-28-e70079-s002.zip › 7.GSEA analysis/5.RNF217-AS1/KEGG_ACUTE_MYELOID_LEUKEMIA.html]

Details for gene set KEGG\_ACUTE\_MYELOID\_LEUKEMIA[GSEA]

|  || Dataset | RNF217-AS1.group.cls#h\_versus\_l |
| Phenotype | group.cls#h\_versus\_l |
| Upregulated in class | l |
| GeneSet | KEGG\_ACUTE\_MYELOID\_LEUKEMIA |
| Enrichment Score (ES) | -0.33904704 |
| Normalized Enrichment Score (NES) | -1.4137256 |
| Nominal p-value | 0.08316008 |
| FDR q-value | 0.35169575 |
| FWER p-Value | 0.852 |
Table: GSEA Results Summary

  

Fig 1: Enrichment plot: KEGG\_ACUTE\_MYELOID\_LEUKEMIA      
 Profile of the Running ES Score & Positions of GeneSet Members on the Rank Ordered List

  

| SYMBOL | TITLE | RANK IN GENE LIST | RANK METRIC SCORE | RUNNING ES | CORE ENRICHMENT || 1 | PIK3R5 | na | 1058 | 0.434 | 0.0465 | No |
| 2 | IKBKB | na | 4368 | 0.238 | -0.0067 | No |
| 3 | BRAF | na | 5517 | 0.199 | -0.0045 | No |
| 4 | PIK3CD | na | 6778 | 0.159 | -0.0126 | No |
| 5 | CCNA1 | na | 7846 | 0.132 | -0.0201 | No |
| 6 | SPI1 | na | 8095 | 0.125 | -0.0050 | No |
| 7 | PPARD | na | 8362 | 0.120 | 0.0085 | No |
| 8 | SOS1 | na | 9467 | 0.096 | -0.0063 | No |
| 9 | PIK3CB | na | 11439 | 0.064 | -0.0519 | No |
| 10 | KRAS | na | 11445 | 0.064 | -0.0407 | No |
| 11 | MTOR | na | 11752 | 0.059 | -0.0391 | No |
| 12 | TCF7L2 | na | 12940 | 0.043 | -0.0658 | No |
| 13 | MAPK1 | na | 13362 | 0.038 | -0.0712 | No |
| 14 | PIK3CA | na | 14021 | 0.031 | -0.0847 | No |
| 15 | MAPK3 | na | 14127 | 0.029 | -0.0826 | No |
| 16 | RARA | na | 14861 | 0.021 | -0.1001 | No |
| 17 | JUP | na | 15702 | 0.012 | -0.1223 | No |
| 18 | AKT2 | na | 15816 | 0.011 | -0.1237 | No |
| 19 | HRAS | na | 15878 | 0.010 | -0.1236 | No |
| 20 | PIK3R1 | na | 16145 | 0.007 | -0.1300 | No |
| 21 | RPS6KB2 | na | 16186 | 0.007 | -0.1299 | No |
| 22 | MAP2K2 | na | 16236 | 0.006 | -0.1302 | No |
| 23 | BAD | na | 16311 | 0.006 | -0.1313 | No |
| 24 | TCF7L1 | na | 16424 | 0.004 | -0.1338 | No |
| 25 | CHUK | na | 16613 | 0.003 | -0.1387 | No |
| 26 | NRAS | na | 17304 | -0.005 | -0.1578 | No |
| 27 | PIM1 | na | 17425 | -0.006 | -0.1603 | No |
| 28 | RPS6KB1 | na | 17704 | -0.008 | -0.1668 | No |
| 29 | STAT5A | na | 18043 | -0.011 | -0.1746 | No |
| 30 | AKT1 | na | 18280 | -0.013 | -0.1790 | No |
| 31 | EIF4EBP1 | na | 18305 | -0.014 | -0.1773 | No |
| 32 | CEBPA | na | 18365 | -0.014 | -0.1765 | No |
| 33 | PML | na | 18765 | -0.018 | -0.1848 | No |
| 34 | CCND1 | na | 18820 | -0.019 | -0.1831 | No |
| 35 | RAF1 | na | 18969 | -0.020 | -0.1839 | No |
| 36 | SOS2 | na | 19710 | -0.027 | -0.2004 | No |
| 37 | TCF7 | na | 19939 | -0.029 | -0.2018 | No |
| 38 | NFKB1 | na | 20531 | -0.035 | -0.2126 | No |
| 39 | IKBKG | na | 20686 | -0.037 | -0.2105 | No |
| 40 | RELA | na | 21550 | -0.046 | -0.2273 | No |
| 41 | GRB2 | na | 22374 | -0.055 | -0.2414 | No |
| 42 | STAT3 | na | 22707 | -0.058 | -0.2406 | No |
| 43 | PIK3R2 | na | 22894 | -0.061 | -0.2352 | No |
| 44 | STAT5B | na | 23102 | -0.063 | -0.2300 | No |
| 45 | MAP2K1 | na | 23215 | -0.064 | -0.2219 | No |
| 46 | ARAF | na | 25299 | -0.095 | -0.2650 | No |
| 47 | RUNX1 | na | 26047 | -0.108 | -0.2674 | No |
| 48 | FLT3 | na | 27793 | -0.143 | -0.2923 | No |
| 49 | KIT | na | 28047 | -0.150 | -0.2731 | No |
| 50 | PIK3CG | na | 30335 | -0.218 | -0.3005 | Yes |
| 51 | MYC | na | 30784 | -0.234 | -0.2720 | Yes |
| 52 | AKT3 | na | 31832 | -0.281 | -0.2523 | Yes |
| 53 | RUNX1T1 | na | 32550 | -0.328 | -0.2149 | Yes |
| 54 | PIM2 | na | 32816 | -0.346 | -0.1611 | Yes |
| 55 | PIK3R3 | na | 32895 | -0.352 | -0.1009 | Yes |
| 56 | ZBTB16 | na | 33262 | -0.381 | -0.0440 | Yes |
| 57 | LEF1 | na | 34024 | -0.487 | 0.0203 | Yes |
Table: GSEA details [plain text format]

  

Fig 2: KEGG\_ACUTE\_MYELOID\_LEUKEMIA      
 Blue-Pink O' Gram in the Space of the Analyzed GeneSet

  

Fig 3: KEGG\_ACUTE\_MYELOID\_LEUKEMIA: Random ES distribution      
 Gene set null distribution of ES for **KEGG\_ACUTE\_MYELOID\_LEUKEMIA**
